# Novel diagnostics for improved treatment of gynecological cancer

**DOI:** 10.48101/ujms.v130.12111

**Published:** 2025-02-13

**Authors:** Ulf Gyllensten

**Affiliations:** Department of Immunology, Genetics and Pathology, Uppsala University, Uppsala, Sweden

**Keywords:** Gynecological cancer, cervical cancer, ovarian cancer, endometrial cancer, proteomics, biomarkers

## Abstract

This paper summarizes the efforts to develop novel biomarkers for diagnosis and screening of the three main gynecological cancers, cervical, endometrial, and ovarian cancer, with an emphasis on research performed during the last 20 years in Uppsala. A cervical cancer screening program has existed in Sweden since 1966 using cytology as the primary test. Over the last two decades, research has provided the scientific base for a transition to self-sampling to improve convenience of the woman and achieve higher population coverage, and use of human papillomavirus as the primary test. Also, efficient prophylactic vaccines and more efficient treatment strategies of women with cervical dysplasia have been introduced. Together, these medical tools have the potential to eradicate cervical cancer by 2120, as envisaged by WHO. By contrast, efficient biomarkers for endometrial and ovarian cancer are still lacking. Through the use of high-throughput proteomics, we have identified novel plasma protein biomarkers to be used in the diagnosis of women with adnexal ovarian mass upon transvaginal ultrasound, and possibly also for early detection in population screening. Similarly, novel biomarkers for the diagnosis of endometrial cancer are being evaluated. To establish a population-based screening program requires careful cost-benefit analyses. One alternative would be to broaden the focus of the current cervical cancer screening program to include also the novel biomarkers for ovarian and endometrial cancer, and thereby achieve screening for all three gynecological cancers. A program that screens for all three diseases could increase motivation to participate and thereby population coverage.

**Figure:**
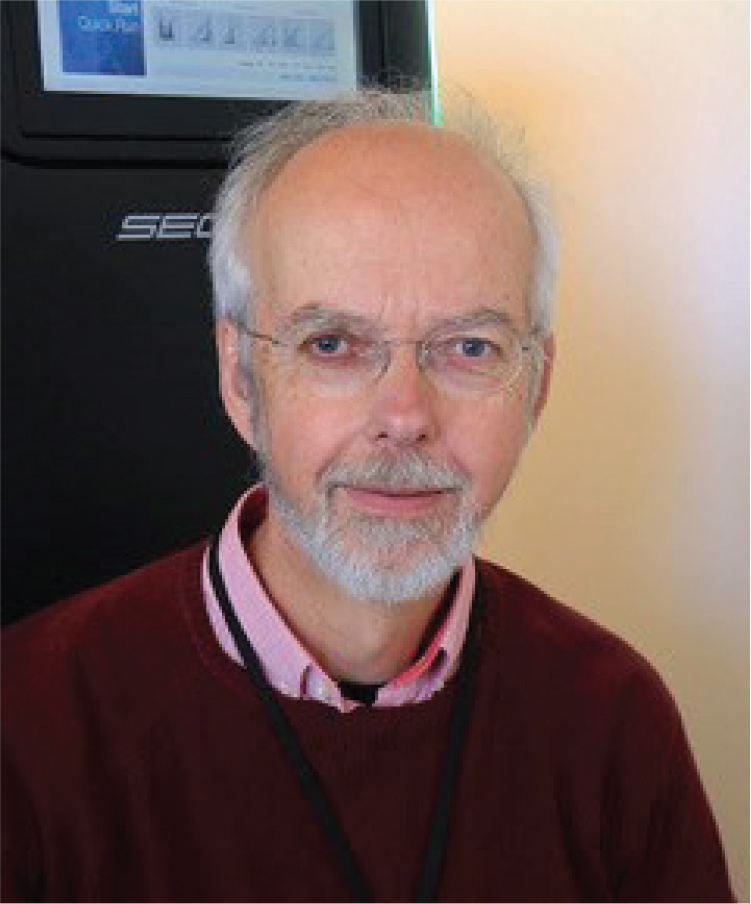
Professor Ulf Gyllensten, winner of the Medical Faculty of Uppsala University Rudbeck Award 2024.

## Background

Gynecological cancer, including cervical, endometrial, and ovarian cancer, represents a significant global health challenge, impacting millions of women each year. Cervical cancer, caused by persistent infection with oncogenic forms of human papillomavirus (HPV), is the fourth most common cancer among women worldwide. In 2022, an estimated 660,000 new cases of cervical cancer were diagnosed, and approximately 350,000 women lost their lives to this preventable disease (1). Endometrial cancer is the most common malignancy of the uterus, and also a growing concern, with approximately 420,000 new cases reported globally in 2022 (2). This cancer predominantly affects postmenopausal women and has been linked to factors such as obesity, hormone replacement therapy, and metabolic syndrome. While survival rates are generally favorable when endometrial cancer is detected early, the increasing prevalence of obesity worldwide has contributed to a rise in the number of cases. Present efforts to address endometrial cancer include promoting a healthy lifestyle, improving early detection through awareness and advancing treatment options for more aggressive forms of the disease.

Ovarian cancer, often referred to as the ‘silent killer’ due to its subtle symptoms and late-stage diagnosis, remains a particularly deadly form of gynecological cancer. In 2012, approximately 239,000 new cases and 152,000 deaths were recorded globally, with incidence rates projected to rise 55% by 2035 (3). This cancer is challenging to detect early, as symptoms like bloating, pelvic pain, and changes in appetite are non-specific and easily overlooked. Survival rates are also significantly lower than for cervical and endometrial cancers, emphasizing the need for enhanced diagnostic tools, increased awareness, and improved therapeutic intervention. Population-based screening represents the only way by which the incidence of ovarian cancer could be reduced, but there is a lack of biomarkers with sufficient sensitivity and specificity to justify screening. Together, these cancers impose a substantial burden on women’s health, highlighting the importance of targeted research, prevention, and management initiatives.

In general, cancer is a chronic disease, and efficient management requires the use of innovative clinical tools at several stages. At the time when symptoms appear, there is a need for precise diagnosis for stratification on alternative follow-up or treatment modalities. After treatment, there needs to be means for efficient monitoring, to detect signs of relapse early. Finally, the only way to effectively reduce the incidence of cancer in the population is either by preventative prophylactic treatment, such as the vaccination program against HPV, or by implementing population-based screening to detect early-stage cancer, such as against cervical cancer, breast cancer, and colorectal cancer. The last approach is feasible only when high-performance, cost-effective diagnostic tests are available.

## Cervical cancer – from etiology to screening and vaccination

The Papanicolaou test, commonly known as the Pap smear test, was developed in the 1920s by Dr. Georgios Papanicolaou, and revolutionized early detection of cervical cancer by enabling microscopic examination of exfoliated cells from the cervix for precancerous and cancerous changes. It was introduced as a cervical cancer screening tool in the 1940s and quickly gained wide acceptance due to its effect on reducing cervical cancer incidence and mortality. The organized screening program in Sweden was initiated in 1966 and based on the Pap smear test.

Pap-smear cytology screening, however, has limited sensitivity, resulting in that women with early-stage cancer may go undetected. Since, in general, cervical cancer has a slow progression rate, the lack of sensitivity has been handled by repeating the screening at frequent intervals. In 1976, Harald zur Hausen provided evidence of an association between HPV and cervical cancer, identifying HPV as the cause of cervical cancer (4). Infection with oncogenic subtypes of HPV disrupts normal cell cycle regulation, which may lead to malignant transformation. This finding refocused the screening to target HPV and initiated primary prevention measures through development of prophylactic vaccines. Zur Hausen received the Nobel Prize in Physiology or Medicine in 2008. However, the journey from identifying HPV as the cause of cervical cancer to implementing HPV testing has been long and complex.

### HPV as a primary screening test

HPV testing was first introduced in Uppsala in 2002 as a triage test for difficult-to-classify cellular abnormalities (ASCUS) and later for postmenopausal women for whom cytology is more challenging to interpret (5, 6). The Uppsala strategy proved to yield excellent results, and in 2010, Swedish national guidelines were established for the use of HPV testing as a triage for ASCUS and cervical intraepithelial neoplasia (CIN1). Uppsala was a pioneer in this first clinical application of HPV testing.

### Self-sampling for women not attending screening

A problem in organized cervical cancer screening programs is poor participation, and although invitations are sent out annually, around 20% of the women do not attend screening. As a possible way to increase the participation rate, we decided to offer women to collect a vaginal sample at her own convenience and send it for HPV testing. In a first pilot project, women who had not participated in screening during the last 6 years were offered a self-sampling kit, and 40% of the women sized this opportunity and performed self-sampling (7–10). Given this high uptake of self-sampling, a series of projects were performed as a foundation for the use of self-sampling in cervical cancer screening.

### HPV genotyping system for self-sampling

A sensitive HPV test was developed, denoted *HPVIR*, which was developed specifically for the analysis of self-collected samples. This test provides more information about the different HPV types present in a sample as compared to most commercial tests. *HPVIR* showed equivalent sensitivity and specificity to the commercial ‘gold-standard’ test at the time, the Hybrid-Capture (*HC2*) (11–14). The *HPVIR* test was subsequently used in Uppsala for the analysis of clinical samples from 2008, with approximately 50,000 samples from the cervical cancer screening program analyzed. *HPVIR* was also validated against the Roche Cobas HPV test for primary screening of women over 30 years old, in accordance with international guidelines for comparing clinical sensitivity and specificity between an approved test (*Cobas HPV test*) and a candidate test (*HPVIR*). *HPVIR* proved to have superior sensitivity and specificity compared to the Cobas HPV test, making it suitable for clinical use either in primary screening or as a follow-up HPV test (15).

### Matrix for self-sampling of vaginal fluid

Uppsala was also pioneering use of a simple dry sampling card Flinders Technology Associates (FTA card) for self-sampling of vaginal fluid in cervical cancer screening. This sample matrix offers several advantages: 1) women can verify that the sample collection has been successful, and material has been deposited on the card through a visible color indication, 2) the cellular material is stable at room temperature, and 3) the sample card can be sent to the HPV laboratory in an envelope via postage (16, 17). This sampling method has been tested and spread to various parts of the world (18–21). For example, a successful project was performed in collaboration with Cape Town University in South Africa, to introduce our system for self-sampling and the *HPVIR* test in rural healthcare centers (22).

### Accuracy of self-sampling versus sampling by healthcare personnel

We performed a series of study to validate self-sampling as a routine method. In the first study, we demonstrated that a vaginal self-sample collected on an FTA card is as informative as a sample taken by healthcare personnel from the cervix or a cervical biopsy, for detecting high-risk HPV. The results showed very good agreement between the self-sample and the samples collected by healthcare personnel, indicating that self-sampling does not reduce sensitivity for detecting an HPV infection (17, 23).

As a follow-up, we conducted a large randomized study involving a total of 19,000 women, half of whom were sampled by a midwife and the other half using self-sampling for HPV testing (24). The results show that self-sampling is as effective as using samples collected by medical professionals for detecting HPV and, during the follow-up, severe dysplasia. (23). All the women in this study were over 50 years old.

### Effect of menstrual cycle on HPV test results

For self-sampling to be conveniently used on a large scale, the results must be consistent across the menstrual cycle. By analyzing samples from a group of women who performed self-sampling every day for a month (excluding days with bleeding), we demonstrated that self-sampling provides reliable HPV results regardless of the day the sample is collected (25).

### Age range of women invited to screening

We had previously demonstrated that HPV testing in primary screening for women above the age of 50 can effectively identify those at high risk of developing cervical dysplasia, while cytology for this age group is less reliable (6). Based on these results, the Uppsala County Council decided to offer all women over 50 the option of HPV testing instead of cytology from 2013. However, about 30% of cervical cancer cases in Sweden occur in women over 60 years old, who, at the time, were no longer included in the screening program. To address this, we performed a study including 1,000 women in the ages from 60 to 75 years old, who were offered self-sampling and HPV testing. The participation rate among those invited was 59.5%. Of these participants, 4.4% tested HPV-positive in their initial test. Dysplasia was detected in 1.8% of the women, with 1.0% having CIN2+. Notably, 81.2% of women with dysplasia had normal results when analyzed using cytology (26, 27). The results showed that self-sampling is an efficient way of screening for cervical cancer also in these age group and emphasized the need to remove the upper age limit of women to be included in the cervical cancer screening program.

### Health-economic benefits of self-sampling

To provide a scientific basis for decisions regarding the screening strategy, we performed a comprehensive health-economic modeling of alternative screening strategies to determine which offers the greatest benefits. This modeling considered all aspects of screening, including both healthcare and individual efforts. The results showed that the screening strategy developed in Uppsala, involving self-sampling and repeated HPV testing, is optimal and provides significant advantages compared to alternative screening strategies (28). An additional health-economic study was recently conducted based on the results from our randomized trial (paragraph below) and provided further evidence that self-sampling and repeated HPV testing are the superior strategies for preventing cervical cancer (29).

### Strategies for follow-up of HPV positive women

One of the criticisms against the use of HPV as the primary test in cervical cancer screening has been that HPV is common in the population (about 5–7% of women in the screening age interval usually test positive), but only a fraction of the women infected will be at risk of cervical cancer. As a consequence, screening using HPV generates a large number of women who need follow-up. The HPV test, thus, has high sensitivity but a lower specificity. Several strategies have been proposed for the follow-up of HPV positive women, including using co-testing, using cytology, stratifying women on HPV genotype (with special concern given to HV16 and HPV18), and use of methylation markers. In each of these strategies, colposcopy and histology based on biopsy would be used for the final diagnosis.

To address the management of HPV positive women, we first proposed a strategy with repeat testing of women who were HPV in their screening test. We showed that the titer of oncogenic HPV is a predictor of risk of cervical dysplasia before any sign of abnormal cytology (30, 31). Further studies demonstrated that HPV viral load could be used to stratify HPV positive patients for follow-up management (32). Since most HPV infections are both asymptomatic and transient, we proposed that a second strategy would be to have women who were positive in their primary screening test simply repeat the HPV test after about 1–2 months, and thereby identify those with a persistent HPV infection. Our first study showed that about 40% of the women cleared their infection between the time of the primary test and the repeat HPV test, and those could be referred back to the screening without need of any clinical management (33).

To further evaluate this screening strategy, we conducted a large randomized study comparing cytology-based screening with self-sampling and repeating for HPV. The study involved 36,400 women aged 30–49 years (34). These women were randomized into two groups: half were offered Pap cytology as a primary test, while the other half performed self-sampling for HPV testing. The participation rate among those offered self-sampling combined with HPV testing was 47% compared to 39% for cytology. Among those who took a self-sample, 6.3% tested HPV-positive in the initial test. Of these, the vast majority (90%) underwent a follow-up test after 4–6 months (average 4.4 months). At the second test, 4.4% of the women were still HPV-positive. The results showed that the repeat HPV testing strategy identified twice as many women with CIN2+ (cervical dysplasia), and with a much shorter turnaround time than the routine strategy at the time (cytology) (34). The results highlight the potential for significant improvement in screening outcomes by selecting an optimized follow-up test strategy.

Self-sampling is today regarded as the most effective way of reducing the cost of screening as well as increasing the population coverage and empowering women to take charge of their own sexual health. From 2022, WHO recommends self-sampling as the default strategy for cervical cancer screening, as part of their effort to promote self-care initiatives in human health (35). In Sweden, self-sampling had been introduced in some regions.

Globally, HPV screening has become a cornerstone in the prevention of cervical cancer, with many countries adopting HPV testing as a primary screening method due to its higher sensitivity compared to cytology. Recommendations for cervical screening exist in 139 (69%) of 202 countries and territories (36, 37). Cytology is the primary screening test in 109 (78%) of 139 countries, while 48 (35%) of 139 countries recommended primary HPV-based screening.

The coverage of HPV screening varies significantly across different regions of the world, influenced by factors such as healthcare infrastructure, economic resources, and public health policies. In high-income countries, organized cervical cancer screening programs are more prevalent, often utilizing Pap smears or HPV DNA tests, leading to higher screening coverage and early detection rates. For instance, countries like the United States, the United Kingdom, and Australia have well-established screening protocols that have contributed to a decline in cervical cancer incidence. In contrast, low- and middle-income countries face challenges in implementing widespread screening due to limited resources, lack of trained healthcare professionals, and insufficient healthcare infrastructure. As a result, screening coverage in these regions remains low, contributing to higher cervical cancer incidence and mortality rates.

The second leg in the prevention of cervical cancer is HPV vaccination. Despite the availability of effective HPV vaccines and screening programs, disparities in access means the burden remains disproportionately high in low- and middle-income countries, where over 85% of cases and deaths occur. These statistics underscore the critical need for comprehensive public health strategies to reduce cervical cancer incidence and mortality globally (38, 39).

The global status and coverage of HPV vaccination exhibit significant disparities across regions, influenced by factors such as healthcare infrastructure, economic resources, and public health policies. Many high-income nations have integrated HPV vaccines into their national immunization programs, achieving substantial coverage rates. As of November 2024, at least 144 countries (approximately 74% of WHO member states) have included the HPV vaccine in their national schedules for girls, with 47 countries (24% of WHO member states) also vaccinating boys (40). In contrast, low- and middle-income countries face challenges such as limited healthcare infrastructure and financial constraints, leading to lower vaccination rates. In 2023, UNICEF supplied HPV vaccines to 52 countries, with seven introducing the vaccine for the first time. However, globally, only about one in eight girls is vaccinated against HPV, indicating significant gaps in coverage (41). The COVID-19 pandemic further exacerbated these disparities, slowing the introduction of HPV vaccines in several regions. A study noted that HPV vaccination coverage among girls in low- and middle-income countries decreased from 65% before the pandemic to 50% during 2020–2021 (42). In summary, while progress has been made in implementing HPV vaccination globally, significant disparities remain.

## Biomarkers for ovarian and endometrial cancers

In contract to cervical cancer, the etiology of both endometrial and ovarian cancers is more complex and less well understood. Also, the biomarkers available have suboptimal sensitivity and/or specificity. Ovarian cancer discovery is mainly symptom-driven or detected through incidental findings (43), and there are no biomarkers available that could justify general screening (44). Ovarian cancer is most commonly diagnosed at late-stage, which leads to an overall 5-year survival of only 30–50%. Patients with spread cancers are detected in stage III or IV and have a 5-year survival rate of less than 30% (45). By contrast, if the cancer could be detected at early stage (stage I), close to 90% of the patients could be cured. Few molecular biomarkers are clinically available to complement imaging, and none meets the accuracy required for screening or reliable diagnosis in symptomatic women (46).

When the trans-vaginal ultrasound (TVU) used for diagnostic imaging indicates ovarian adnexal mass, diagnostic surgery with curative intention is used to confirm indications by TVU. In Sweden, about 75% of the women with adnexal ovarian mass and evaluated by diagnostic surgery have benign ovarian tumors (benign cysts) (47). Surgical intervention, by itself, is not risk-free, and surgery-related complications, such as side-effects on fertility and induced menopause, have been reported in between 3.5% to as high as 15% of the women with benign ovarian adnexal mass (48). Imaging techniques can separate benign from malignant conditions, but the experience of the medical personnel impacts the performance and the cost-benefit for the healthcare system (49).

A biomarker test that accurately distinguishes between women with benign and malignant changes could reduce the referrals to tertiary centers and reduce the need for surgical intervention and its side-effects. MUCIN-16 (CA-125) is currently the best biomarker in ovarian cancer diagnosis of post-menopausal women (50). However, MUCIN-16 has low sensitivity for the detection of early-stage ovarian cancer, and high levels can be found in benign gynecological conditions, such as infections, pregnancies, and endometriosis (50, 51). MUCIN-16 has also been elevated in women with acute pancreatitis and some elderly women with heart failures (52, 53). The performance of MUCIN-16 can be enhanced by combining it with biomarkers such as WAP Four-Disulfide Core Domain 2 (WFDC2 or HE4) as in the ROMA Score (Ovarian Malignancy Risk Algorithm). The ROMA score has been reported to have an overall sensitivity of 87.0% at a specificity of 80.9% in pre-menopausal and 91.1 and 77.2% in post-menopausal women, respectively (53). By using several protein biomarkers in the test, the performance can be improved. The OVA1-test, for example, combines five proteins (Apolipoprotein A1, Beta 2 microglobulin, MUCIN-16, Transferrin and Prealbumin/Transthyretin) and is used to divide women into groups with high, intermediate, or low risk of ovarian cancer. In the OVERA-test, five proteins (MUCIN16, Transferrin, Apolipoprotein A1, Follicle-stimulating hormone, and WFDC2) are included, and this test has been reported to have a sensitivity of 69% at a specificity of 91% (54).

We have previously developed (55) and validated (56) a multiplex biomarker panel with 11 proteins (MUCIN-16, SPINT1, TACSTD2, CLEC6A, ICOSLG, MSMB, PROK1, CDH3, WFDC2, KRT19, and FR-alpha) plus individual age, which has higher ability than MUCIN-16 to distinguish between women with benign and malignant conditions at the time of diagnosis. In two independent validation cohorts with both pre- and post-menopausal women, our 11-protein panel achieved sensitivities of 83–88% at specificities of 88–92% for a pre-defined cut-off (56). Based on the 11-protein panel, we developed a risk-score between 0 and 1 for distinguishing between benign tumors and ovarian cancer in women with adnexal ovarian mass. The performance of the risk-score model was validated and showed indistinguishable performance in two independent Swedish clinical cohorts. Over 90% of the women with benign tumors in the two validation cohorts had risk-scores below the cut-off for malignancy and would therefore not have needed diagnostic surgery. In comparison with clinically measured MUCIN-16 (CA-125), our risk-score showed a higher specificity at a retained sensitivity for the classification of benign tumors at the time of diagnosis.

We also examined the ovarian cancer risk-score based on the 11-proteins at different stages during the clinical course of the disease and cancer treatment, using samples collected from before treatment initiation, during treatment, after completion of treatment, and during relapse (56). The risk-score dropped during treatment and continued to do so in patients responding to treatment. After completion of treatment, the risk-score started to increase but at different rates in individual women. Taken together, the risk-score followed the clinical course of the disease and the treatment outcome.

When following the development from diagnosis and during the 2-year follow-up period for individual women, we identified four main risk-score trajectories (56). These trajectories correspond to four common clinical responses, such as 1) lack of treatment response, 2) good initial treatment response followed by rapid relapse, 3) good treatment response and slow increase in relapse risk, and finally, 4) good treatment response and low mortality during follow-up. We also showed that the risk-score at diagnosis is, second to tumor stage based on histology, the strongest predictor of 5-year survival. In the same analysis, clinically measured MUCIN-16 at the time of diagnosis did not show any statistically significant predictivity (56). We have expanded the number of proteins studied using the Olink panels to include 1,536 plasma proteins (57), followed by 3,072 plasma proteins (58) and most recently analyzed a total of 5,300 plasma proteins (unpublished), in order to search for additional biomarker candidates for ovarian and endometrial cancer. Studies of a range of cancers, including gynecological cancers discussed here, have shown that large-scale screening of the plasma proteome represents a promising route for the identification of biomarker candidates for many types of cancer (59). These technologies are also readily applied to self-collected fingerstick blood or vaginal fluid, facilitating their use in future population screening or monitoring for relapse during follow-up (60).

For endometrial cancer, there is also a lack of biomarkers to aid in the diagnosis of patients. In management of women with diffuse symptoms or post-menopausal bleeding, there is a need to distinguish between type 1 endometrioid cancers, associated with high estrogen and less aggressive, and type 2 endometrial cancer, which is more likely to spread outside the uterus and has a worse outlook, as well as from the differential diagnosis of endometriosis. Analyses are underway using the high-throughput proteomics technology described to identify suitable biomarkers also of endometrial cancer (unpublished).

## Diagnosis and screening of gynecological cancer – a vision for the future

The main focus of the use of the novel protein biomarkers has been to improve diagnosis of women with adnexal ovarian mass, and in the future, for women with post-menopausal bleeding at risk of endometrial cancer. To reduce the incidence of ovarian cancer, however, it is necessary to develop tests that can be used for the identification of early-stage cancer in asymptomatic women in screening. The dramatic reduction of our plasma protein-based risk-score in response to treatment and its increase during relapse indicates that the risk-score reflects tumor burden, and that it may potentially inform on risk of future disease also before the development of symptoms. Further studies are in the process to evaluate its potential in population-screening.

In general, screening could be in the form of annual or biannual testing of the high-risk age strata of women. If this is carried out using self-sampling, burdening of primary health-care centers with routine sample collection could be avoided. Such longitudinal sample collection in the risk-group would also enable the use of individual risk-score thresholds, based on trend analyses of personalized levels, for the identification of early-stage ovarian cancer, which could be more sensitive than using general population-based thresholds (61). Even if suitable biomarkers for population screening for ovarian and endometrial cancer were available, establishing a screening program would have to justify from health-economic perspective. Given that gynecological cancers are not among the most prevalent types of cancer, there would be justified concerns. An alternative would be to broaden the focus of the existing cervical cancer screening program to include all three gynecological cancers. Since the cervical cancer screening program is already ongoing, including additional biomarkers from the samples collected and possibly adding a clinical sample type such as fingerstick blood would be more feasible.

**Figure 1:**
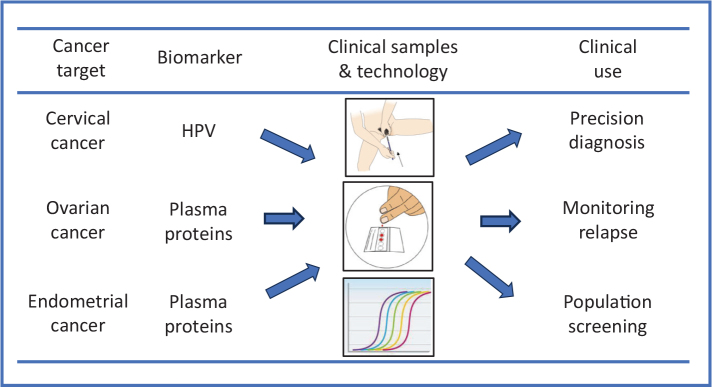
Schematic outline of a common screening strategy for gynecological cancers.

Developing a joint screening program for cervical, endometrial, and ovarian cancers would be a groundbreaking step in gynecological health but requires careful planning, significant investment, and collaboration across medical and policy domains. Based on the existing technologies and present understanding of the diagnostics targets (i.e. HPV, plasma proteins, and possibly circulating tumor DNA [ctDNA]), integrating multiple targets into a single assay would be feasible, given that the detection of both HPV DNA (and possibly also other DNA targets) and plasma proteins using the Proximity Extension Assay (PEA) can all be achieved using a single read-out technology, such as real-time PCR. A schematic outline of such screening test is shown in Figure 1.

The fact that the screening would target several cancers might increase motivation for women to participate. The pros of such a joint screening program for cervical, endometrial, and ovarian cancers include 1) integrated prevention, potentially reducing morbidity and mortality, 2) early detection of cancers that currently lack screening programs, 3) efficient use of existing infrastructure, and 4) multiple cancer screening might encourage higher participation. However, here are also a number of cons to consider, 1) challenges in integrating different test modalities, 2) screening for multiple cancers may lead to overdiagnosis and overtreatment, 3) balancing sensitivity and specificity for three cancers is complex, and 4) different cancers may require separate sample types complicating implementation.

## References

[1] World Health Organization. Cervical cancer: estimated incidence, mortality, and prevalence worldwide in 2022. Available from: https://www.who.int/news-room/fact-sheets/detail/cervical-cancer [cited 2 March 2024].

[2] World Cancer Research Fund. Endometrial cancer statistics. Available from: https://www.wcrf.org/preventing-cancer/cancer-statistics/endometrial-cancer-statistics [cited 2 March 2024].

[3] World Ovarian Cancer Coalition. The World Ovarian Cancer Coalition Atlas 2018. Available from: https://worldovariancancercoalition.org/wp-content/uploads/2018/10/THE-WORLD-OVARIAN-CANCER-COALITION-ATLAS-2018.pdf.

[4] zur Hausen H. Condylomata acuminata and human genital cancer. Cancer Res. 1976;36:794. 175942

[5] de Lang A, Wikström I, Wilander E. Significance of HPV tests on women with cervical smears showing ASCUS. Acta Obstet Gynecol Scand. 2005;84:1001–5. doi: 10.1111/j.0001-6349.2005.00825.x 16167919

[6] Gyllensten U, Gustavsson I, Lindell M, Wilander E. Primary high-risk HPV screening for cervical cancer in post-menopausal women. Gynecol Oncol. 2012;125:343–5. doi: 10.1016/j.ygyno.2012.01.036 22293044

[7] Stenvall H, Wikström I, Backlund I, Wilander E. Accuracy of HPV test of vaginal smear obtained with a novel self-sampling device. Acta Obstet Gynecol Scand. 2007;86:16–21. doi: 10.1080/00016340601033667 17230283

[8] Sanner K, Wikström I, Strand A, Lindell M, Wilander E. Self-sampling of the vaginal fluid at home combined with high-risk HPV testing. Br J Cancer. 2009;101:871–4. doi: 10.1038/sj.bjc.6605194 19654577 PMC2736844

[9] Lindell M, Sanner K, Wikström I, Wilander E. Self-sampling of vaginal fluid and high-risk human papillomavirus testing in women aged 50 years or older not attending Papanicolaou smear screening. BJOG. 2012;119:245–8. doi: 10.1111/j.1471-0528.2011.03147.x 22017806

[10] Wikström I, Lindell M, Sanner K, Wilander E. Self-sampling and HPV testing or ordinary Pap-smear in women not regularly attending screening: a randomised study. Br J Cancer. 2011;105:337–9. doi: 10.1038/bjc.2011.236 21730977 PMC3172898

[11] Josefsson A, Livak K, Gyllensten U. Detection and quantitation of human papillomavirus by using the fluorescent 5’ exonuclease assay. J Clin Microbiol. 1999;37:490–6. doi: 10.1128/JCM.37.3.490-496.1999 9986801 PMC84442

[12] Ylitalo N, Bergström T, Gyllensten U. Detection of genital human papillomavirus by single-tube nested PCR and type-specific oligonucleotide hybridization. J Clin Microbiol. 1995;33:1822–8. doi: 10.1128/jcm.33.7.1822-1828.1995 7665652 PMC228277

[13] Moberg M, Gustavsson I, Gyllensten U. Real-time PCR-based system for simultaneous quantification of human papillomavirus types associated with high risk of cervical cancer. J Clin Microbiol. 2003;41:3221–8. doi: 10.1128/JCM.41.7.3221-3228.2003 12843067 PMC165384

[14] Gustavsson I, Juko-Pecirep I, Backlund I, Wilander E, Gyllensten U. Comparison between the Hybrid Capture 2 and the hpVIR real-time PCR for detection of human papillomavirus in women with ASCUS or low-grade dysplasia. J Clin Virol. 2009;45:85–9. doi: 10.1016/j.jcv.2009.04.012 19451022

[15] Gustavsson I, Aarnio R, Myrnäs M, Hedlund-Lindberg J, Taku O, Meiring T, et al. Clinical validation of the HPVIR high-risk HPV test on cervical samples applied on the FTA card according to the international guidelines for human papillomavirus DNA test requirements for cervical screening. Virol J. 2019;16:107. doi: 10.1186/s12985-019-1216-7 31438976 PMC6704622

[16] Gustavsson I, Lindell M, Wilander E, Strand A, Gyllensten U. Use of FTA card for dry collection, transportation, and storage of cervical cell specimen to detect high-risk HPV. J Clin Virol. 2009;46:112–6. doi: 10.1016/j.jcv.2009.06.021 19628427

[17] Gustavsson I, Sanner K, Lindell M, Strand A, Olovsson M, Wikström I, et al. Type-specific detection of high-risk human papillomavirus (HPV) in self-sampled cervicovaginal cells applied to FTA elute cartridge. J Clin Virol. 2011;51:255–8. doi: 10.1016/j.jcv.2011.05.006 21632283

[18] Maurer K, Luo H, Shen Z, Wang G, Du H, Wang C, et al. Evaluation of a new solid media specimen transport card for high-risk HPV detection and cervical cancer prevention. J Clin Virol. 2016;76:14–9. doi: 10.1016/j.jcv.2015.12.010 26774544

[19] Catarino R, Vassilakos P, Bilancioni A, Vanden Eynde M, Meyer-Hamme U, Menoud PA, et al. Randomized comparison of two vaginal self-sampling methods for human papillomavirus detection: dry swab versus FTA cartridge. PLoS One. 2015;10:e0143644. doi: 10.1371/journal.pone.0143644 26630353 PMC4668032

[20] Wang SM, Hu SY, Chen W, Chen F, Zhao FH, He W, et al. Feasibility and accuracy evaluation of three human papillomavirus assays for FTA card-based sampling: a pilot study in cervical cancer screening. BMC Cancer. 2015;15:848. doi: 10.1186/s12885-015-1882-9 26537356 PMC4634578

[21] Luo H, Du H, Maurer K, Belinson JL, Wang G, Liu Z, et al. An evaluation of the Cobas4800 HPV test on cervico-vaginal specimens in liquid versus solid transport media. PLoS One. 2016;11:e0148168. doi: 10.1371/journal.pone.0148168 26828360 PMC4734716

[22] Taku O, Meiring TL, Gustavsson I, Phohlo K, Garcia-Jardon M, Mbulawa ZZA, et al. Acceptability of self-collection for human papillomavirus detection in the Eastern Cape, South Africa. PLoS One. 2020;15:e0241781. doi: 10.1371/journal.pone.0241781 33170891 PMC7654756

[23] Gustavsson I, Aarnio R, Berggrund M, Hedlund-Lindberg J, Sanner K, Wikström I, et al. Randomised study of HPV prevalence and detection of CIN2+ in vaginal self-sampling compared to cervical specimens collected by medical personnel. Int J Cancer. 2019;144:89–97. doi: 10.1002/ijc.31637 29943822

[24] Aarnio R, Isacson I, Sanner K, Gustavsson I, Gyllensten U, Olovsson M. Comparison of vaginal self-sampling and cervical sampling by medical professionals for the detection of HPV and CIN2+: a randomized study. Int J Cancer. 2021;148:3051–9. doi: 10.1002/ijc.33482 33497465

[25] Sanner K, Wikström I, Gustavsson I, Wilander E, Lindberg JH, Gyllensten U, et al. Daily self-sampling for high-risk human papillomavirus (HR-HPV) testing. J Clin Virol. 2015;73:1–7. doi: 10.1016/j.jcv.2015.09.016 26498105

[26] Lindström A, Sanchez Hermansson R, Gustavsson I, Hedlund-Lindberg J, Gyllensten U, Olovsson M. Cervical dysplasia in elderly women performing repeated self-sampling for HPV testing. PLoS One. 2018;13:e0207714. doi: 10.1371/journal.pone.0207714 30517176 PMC6281203

[27] Hermansson RS, Olovsson M, Gustavsson I, Gyllensten U, Lindkvist O, Lindberg JH, et al. Incidence of oncogenic HPV and HPV-related dysplasia five years after a negative HPV test by self-sampling in elderly women. Infect Agent Cancer. 2022;17:42. doi: 10.1186/s13027-022-00453 35922825 PMC9351123

[28] Östensson E, Hellström AC, Hellman K, Gustavsson I, Gyllensten U, Wilander E, et al. Projected cost-effectiveness of repeat high-risk human papillomavirus testing using self-collected vaginal samples in the Swedish cervical cancer screening program. Acta Obstet Gynecol Scand. 2013;92:830–40. doi: 10.1111/aogs.12143 23530870

[29] Aarnio R, Östensson E, Olovsson M, Gustavsson I, Gyllensten U. Cost-effectiveness analysis of repeated self-sampling for HPV testing in primary cervical screening: a randomized study. BMC Cancer. 2020;20:645. doi: 10.1186/s12885-020-07085-9 32660432 PMC7359275

[30] Ylitalo N, Sørensen P, Josefsson AM, Magnusson PK, Andersen PK, Pontén J, et al. Consistent high viral load of human papillomavirus 16 and risk of cervical carcinoma in situ: a nested case-control study. Lancet. 2000;355:2194–8. doi: 10.1016/S0140-6736(00)02402-8 10881892

[31] Josefsson AM, Magnusson PK, Ylitalo N, Sørensen P, Qwarforth-Tubbin P, Andersen PK, et al. Viral load of human papilloma virus 16 as a determinant for development of cervical carcinoma in situ: a nested case-control study. Lancet. 2000;355:2189–93. doi: 10.1016/S0140-6736(00)02401-6 10881891

[32] Berggrund M, Gustavsson I, Aarnio R, Hedlund-Lindberg J, Sanner K, Wikström I, et al. HPV viral load in self-collected vaginal fluid samples as a predictor for the presence of cervical intraepithelial neoplasia. Virol J. 2019;16:146. doi: 10.1186/s12985-019-1253-2 31771594 PMC6880361

[33] Gyllensten U, Sanner K, Gustavsson I, Lindell M, Wikström I, Wilander E. Short-time repeat high-risk HPV testing by self-sampling for screening of cervical cancer. Br J Cancer. 2011;105:694–7. doi: 10.1038/bjc.2011.277 21811250 PMC3188941

[34] Gustavsson I, Aarnio R, Berggrund M, Hedlund-Lindberg J, Strand AS, Sanner K, et al. Randomised study shows that repeated self-sampling and HPV test has more than twofold higher detection rate of women with CIN2+ histology than Pap smear cytology. Br J Cancer. 2018;118:56–64. doi: 10.1038/bjc.2017.485 PMC5886121 29438367

[35] World Health Organization. Self-care interventions for health: WHO consolidated guideline. Available from: https://www.who.int/publications/i/item/WHO-SRH-23.1 [cited 17 April 2023].

[36] Bruni L, Serrano B, Roura E, Alemany L, Cowan M, Herrero R, et al. Cervical cancer screening programmes and age-specific coverage estimates for 202 countries and territories worldwide: a review and synthetic analysis. Lancet. 2022;10:e1115–27. doi: 10.1016/S2214-109X(23)00240-1 PMC9296658 35839811

[37] World Health Organization. WHO prequalifies additional HPV test expanding options as countries pursue cervical cancer elimination. Available from: https://www.who.int/news/item/14-06-2023-who-prequalifies-additional-hpv-test-expanding-options-as-countries-pursue-cervical-cancer-elimination [cited 14 June 2023].

[38] Spayne J, Hesketh T. Estimate of global human papillomavirus vaccination coverage: analysis of country-level indicators. BMJ Open. 2021;11:e052016. doi: 10.1136/bmjopen-2021-052016 PMC8413939 34475188

[39] Our World in Data. Human papillomavirus vaccine immunization schedule. Available from: https://ourworldindata.org/grapher/human-papillomavirus-vaccine-immunization-schedule [cited 9 Jan 2025].

[40] World Health Organization. WHO adds an HPV vaccine for single-dose use. Available from: https://www.who.int/japan/news/detail-global/04-10-2024-who-adds-an-hpv-vaccine-for-single-dose-use [cited 4 Oct 2024].

[41] UNICEF. Closing the gap: UNICEF bolsters country efforts to increase HPV vaccination. Available from: https://www.unicef.org/supply/stories/closing-gap-unicef-bolsters-country-efforts-increase-hpv-vaccination [cited 25 April 2023].

[42] Casey RM, Akaba H, Hyde TB, Bloem P. Covid-19 pandemic and equity of global human papillomavirus vaccination: descriptive study of World Health Organization-UNICEF vaccination coverage estimates. BMJ Med. 2024;3:e000726. doi: 10.1136/bmjmed-2023-000726 PMC10826539 38293682

[43] Froyman W, Landolfo C, De Cock B, Wynants L, Sladkevicius P, Testa AC, et al. Risk of complications in patients with conservatively managed ovarian tumours (IOTA5): a 2-year interim analysis of a multicentre, prospective, cohort study. Lancet Oncol. 2019;20:448–58. doi: 10.1016/S1470-2045(18)30837-4 30737137

[44] Menon U, Gentry-Maharaj A, Burnell M, Singh N, Ryan A, Karpinskyj C, et al. Ovarian cancer population screening and mortality after long-term follow-up in the UK Collaborative Trial of Ovarian Cancer Screening (UKCTOCS): a randomised controlled trial. Lancet. 2021;397:2182–93. doi: 10.1016/S0140-6736(21)00731-5 33991479 PMC8192829

[45] Bast RC, Han CY, Lu Z, Lu KH. Next steps in the early detection of ovarian cancer. Commun Med. 2021:1:36. doi: 10.1038/s43856-021-00037-9 34676377 PMC8525879

[46] Menon U, Gentry-Maharaj A, Hallett R, Ryan A, Burnell M, Sharma A, et al. Sensitivity and specificity of multimodal and ultrasound screening for ovarian cancer, and stage distribution of detected cancers: results of the prevalence screen of the UK Collaborative Trial of Ovarian Cancer Screening (UKCTOCS). Lancet Oncol. 2009;10:327–40. doi: 10.1016/S1470-2045(09)70026-9 19282241

[47] Lycke M, Kristjansdottir B, Sundfeldt K. A multicenter clinical trial validating the performance of HE4, CA125, risk of ovarian malignancy algorithm and risk of malignancy index. Gynecol Oncol. 2018;151:159–65. doi: 10.1016/j.ygyno.2018.08.025 30149898

[48] Jacobs IJ, Menon U, Ryan A, Gentry-Maharaj A, Burnell M, Kalsi JK, et al. Ovarian cancer screening and mortality in the UK Collaborative Trial of Ovarian Cancer Screening (UKCTOCS): a randomised controlled trial. Lancet. 2016;387:945–56. doi: 10.1016/S0140-6736(15)01224-6 26707054 PMC4779792

[49] Tian C, Wen SB, Zhao CY, Yan XN, Du JX. Comparative diagnostic accuracy of the IOTA SRR and LR2 scoring systems for discriminating between malignant and benign adnexal masses by junior physicians in Chinese patients: a retrospective observational study. BMC Womens Health. 2023;23:27–9. doi: 10.1186/s12905-023-02719-z 37940895 PMC10633950

[50] Davenport C, Rai N, Sharma P, Deeks JJ, Berhane S, Mallett S, et al. Menopausal status, ultrasound and biomarker tests in combination for the diagnosis of ovarian cancer in symptomatic women. Cochrane Database Syst Rev. 2022;11:CD011964. doi: 10.1002/14651858.CD011964.pub2 PMC9314189 35879201

[51] Sölétormos G, Duffy MJ, Verheijen RH, Tholander B, Bast RC, Gaarenstroom KN, et al. Clinical use of cancer biomarkers in epithelial ovarian cancer: updated guidelines from the European Group on Tumor Markers. Int J Gynecol Cancer. 2016;26:43–51. doi: 10.1097/IGC.0000000000000586 26588231 PMC4679342

[52] Lycke M, Ulfenborg B, Lauesgaard MJ, Kristjansdottir B, Sundfeldt K. Consideration should be given to smoking, endometriosis, renal function (eGFR) and age when interpreting CA125 and HE4 in ovarian tumor diagnostics. Clin Chem Lab Med. 2021;59:1954–62. doi: 10.1515/cclm-2021-0510 34388324

[53] Ding L, Zhou YX, He C, Ai JY, Lan GL, Xiong HF, et al. Elevated CA125 levels are associated with adverse clinical outcomes in acute pancreatitis: a propensity score-matched study. Pancreatology. 2020;20:789–94. doi: 10.1016/j.pan.2020.06.009 32660761

[54] Coleman RL, Herzog TJ, Chan DW, Munroe DG, Pappas TC, Smith A, et al. Validation of a second-generation multivariate index assay for malignancy risk of adnexal masses. Am J Obstet Gynecol. 2016;215:82.e1–11. doi: 10.1016/j.ajog.2016.03.003 26970494

[55] Enroth S, Berggrund M, Lycke M, Broberg J, Lundberg M, Assarsson E, et al. High throughput proteomics identifies a high-accuracy 11 plasma protein biomarker signature for ovarian cancer. Commun Biol. 2019;2:221. doi: 10.1038/s42003-019-0464-9 31240259 PMC6586828

[56] Enroth S, Ivansson E, Lindberg JH, Lycke M, Bergman J, Reneland A, et al. Data-driven analysis of a validated risk score for ovarian cancer identifies clinically distinct patterns during follow-up and treatment. Commun Med. 2022;2:13. doi: 10.1038/s43856-022-00193-6 36196264 PMC9526736

[57] Gyllensten U, Hedlund-Lindberg J, Svensson J, Manninen J, Öst T, Ramsell J, et al. Next-generation plasma proteomics identifies high-precision biomarker candidates for ovarian cancer. Cancers (Basel). 2022;14:1757. doi: 10.3390/cancers14071757 35406529 PMC8997113

[58] Ivansson E, Lindberg JH, Stålberg K, Sundfeldt K, Gyllensten U, Enroth S. Large-scale proteomics reveals precise biomarkers for detection of ovarian cancer in symptomatic women. Sci Rep. 2024;14:17288. doi: 10.1038/s41598-024-68249-2 39068297 PMC11283551

[59] Álvez MB, Edfors F, von Feilitzen K, Zwahlen M, Mardinoglu A, Edqvist PH, et al. Next-generation pan-cancer blood proteome profiling using proximity extension assay. Nat Commun. 2023;14:13. doi: 10.1038/s41467-023-39765-y 37463882 PMC10354027

[60] Lindberg JH, Widgren A, Ivansson E, Gustavsson I, Stålberg K, Gyllensten U, et al. Toward ovarian cancer screening with protein biomarkers using dried, self-sampled cervico-vaginal fluid. iScience. 2024;27:109001. doi: 10.1016/j.isci.2024.109001 38352226 PMC10863317

[61] Russell MR, Lyon J, Balkwill F. Diagnosis of epithelial ovarian cancer using a combined protein biomarker panel. Br J Cancer. 2019;121:483–9. doi: 10.1038/s41416-019-0544-0 31388184 PMC6738042

